# Longitudinal effects of physical activity on self-efficacy and cognitive processing of active and sedentary elderly women

**DOI:** 10.1590/S1980-57642014DN82000016

**Published:** 2014

**Authors:** Sofia Rosanti, Guilherme Elias da Silva, Flávia Heloísa Santos

**Affiliations:** 1 UNESP, Universidade Estadual Paulista, Campus Bauru, SP, Brazil; 2 UEM, Universidade Estadual de Maringá, Maringá PR, Brazil; 3 Universidade do Minho, Braga, Portugal

**Keywords:** aging, physical activity, self-efficacy, episodic memory, praxis

## Abstract

Previous studies support that regular physical activity in aging contributes as a protective factor against cognitive decline and improves mood states. However, there is a lack of longitudinal studies in this area.

**Objective:**

To observe possible changes in cognition related with physical activity.

**Methods:**

This study reassessed, after one-year period, 31 elderly women divided into two groups, sedentary *versus* active, using behavioral scales and cognitive tests.

**Results:**

The active group exhibited significantly enhanced performance in general cognitive function, particularly on tasks of episodic memory and praxis, and also on the mood states scale compared to the sedentary group. The active women also reported higher self-efficacy.

**Conclusion:**

Long-term physical activity promoted improvement on quality of life in the elderly women.

## Abstract (pt)

Estudos anteriores sustentam que a prática regular de exercícios físicos contribui como fator de proteção aos declínios cognitivos e melhoras no estado de humor no envelhecimento. Contudo, há uma carência de estudos longitudinais nesta área.

**Objetivo:**

Observar possíveis alterações na cognição em relação a atividade física.

**Métodos:**

O presente estudo reavaliou após um ano 31 idosas divididas em dois grupos, sedentárias *versus* ativas, por meio de escalas comportamentais e testes cognitivos.

**Resultados:**

Houve um desempenho significativamente melhor em idosas ativas em funções cognitivas gerais, mais especificamente em tarefas da memória episódica e praxia, como também para a escala de estado de humor, em comparação com o grupo de sedentárias. O grupo de ativas também apresentou maior auto eficácia.

**Conclusão:**

Práticas de atividades físicas a longo prazo promoveu melhora na qualidade de vida de idosas.

## INTRODUCTION

The elderly population has been growing every year, both in developed and developing countries.^1^ The life expectancy of the Brazilian population increased from 63 to 73 years between 1990 and 2009,2 with a consequent higher prevalence of chronic-degenerative diseases in this population,^3^ particularly dementia.^4,5^

Biological, psychological and social changes are often observed during the aging process^6-9^ and a significant portion of this population exhibits cognitive deficits, especially memory dysfunctions,^10-12^ affecting episodic memory more than semantic memory.^13^

Cognitive deficits may be strongly associated with depression. The more severe the depression, the greater the cognitive and functional impairment of patients, resulting in impaired quality of life.^14^ The level of education of elderly also influences the extent of decline in cognitive skills.^15^ In Brazil, the average schooling of elderly is 4.1 years for the country as a whole,^16^ whereas 30.7% have less than a year of formal education.^3^

Mnemonic losses may be interpreted by the concept of self-efficacy, which denotes the self-evaluation an individual carries out on their own ability to perform a task within a specific field.^17^ Individuals with high self-efficacy, who feel able to accomplish a particular task, invest greater effort to perform it, have greater motivation to complete it and persevere longer in executing the task than individuals with low self-efficacy. Therefore, perceived self-efficacy exerts a regulatory function on behavior and contributes to the quality of psychosocial functioning.^17^

There is a tendency toward physical inactivity among Brazilians. The percentage of people who practice physical activity regularly is 10.8% and 5.2% among men and women, respectively.^16^ Regular physical activity yields benefits such as higher longevity, improved cardiorespiratory and muscular capacity, aids weight control and nutrition, increases strength, and resistance in general; improves flexibility, coordination, and balance.^18,19^ Besides, it promotes greater self-motivation and sense of self efficacy,^20,21^ preventing the development of chronic diseases and improving the quality of life in aging.^22^ Moreover, regular physical activity can lead to improvement of cognitive functions such as memory, attention, executive functions and praxis,^18,23-25^ thus constituting a protective factor for cognitive impairment in elderly.^26-28^

Nevertheless, there are few studies with longitudinal tracking to help ensure a better quality of life have been conducted in this population.^29-35^ When problems are detected early, there is a greater chance of providing adequate intervention and stable maintenance of these abilities for a longer period of time.^36,37^

The aims of this study were to assess possible changes in neuropsychological profile and to evaluate the concept of self-efficacy in active and sedentary elderly followed over a 12-month period by reassessing these parameters and investigating their relationship with the practice of physical activity.

## METHODS

Participants were 31 elderly women, aged 60-70 years (M=67.1, SD=3.4) with an educational level of 4-15 years (M=6.4 years, SD=3.3), living in the rural area of São Paulo State. Of the study sample, 17 were participants of the *Agita Assis* project - practicing physical activity at least three times a week, with each session lasting at least 50 minutes - and comprised the experimental group. The remaining 14 elderly women were sedentary - not practicing any physical activity - and comprised the control group. The women were assessed by screening and neuropsychological assessment.

### Instruments.

*General Screening* - Anamnesis;^26^ Raven's Progressive Matrices Scale, series A, B, C, D and E.;^38^ CAMCOG - Cognitive Section of the CAMDEX;^39,40^ Brazil Economic Classification Criterion (CCEB);^41^ Geriatric Depression Scale (GDS-SF), shorter version with fifteen items;^42^ AUDIT (Alcohol Use Disorders Identification Test).^43^

*Neuropsychological Assessment* - Free recall of word list;^44^ Corsi block-tapping task;^45^ Phonological verbal fluency task. Letters F, A and S;^46^ Wechsler Adult Intelligence Scale - revised (WAIS-R) - Digit Span subtest;^47^ Wechsler Memory Scale third edition (WMS-III) - subtests: Logical Memory subtest; Visual Reproduction subtest; Vocabulary subtest;^48^ Index of Independence in Activities of Daily Living (ADL).^49^

*Memory Self-Efficacy Questionnaire (MSEQ)*^50^ - This is an instrument for assessing self-efficacy through seven questions based on the execution of memory tasks (for example recall of everyday objects, phone numbers, and names of people in photos) on which participants judge their own ability to achieve them. It should be noted that the memory tasks in this questionnaire mainly involve episodic memory skill. A version of the questionnaire translated and adapted for use in Brazilian samples was applied.^51^

**Statistical analyses.** For inferential purposes, repeated measures ANOVA was carried out considering group as the independent variable (sedentary versus active) and the total score obtained on each test or scale as dependent variables. Also taking into account the effects of time elapsed between the two assessment periods (baseline and after one year), differences were confirmed by the *post hoc* Tukey HDS test. For all comparisons, the level of significance was set at p≤0.05. Effect sizes were obtained by the ETA-squared and classified as: small 0.1, medium 0.6 and large effects 0.14.^52^

**Ethical aspects.** The study was approved by the Research Ethics Committee of the State University of São Paulo State, "Júlio de Mesquita Filho", under case no. 464/2006.

## RESULTS

The results were produced by comparing the performance achieved on tests and scales in the assessment periods by group (active and sedentary), as well as the interaction between the period and performance of each group for each variable evaluated.

The results in Table 1 showed no group effect for total score on the CAMCOG [F (1.29)<0.01, p=0.93] and no effect of assessment period [F (1.29)=1.04, p=0.32]. However, a significant interaction was observed [F (1.29)=7.41, p=0.01].

**Table 1:** Comparison between performances by groups on parameters assessed in the screening at baseline assessment and after one year.

Parameters assessed	Active (n=17)			Sedentary (n=14)		Effects	F	p	η2
	Baseline assessment M±(SD)	After one year M±(SD)		Baseline assessment M±(SD)	After one year M±(SD)				
CAMCOG	87.1 (8.7)	88.1 (8.0)		87.6 (6.8)	87.1 (6.7)	Group	<0.01	0.93	.00
						Period	1.03	0.31	
						Interaction	7.41	0.01*	
Orientation	9.8 (0.4)	9.9 (0.3)		9.7 (0.5)	9.8 (0.4)	Group	0.27	0.60	.02
						Period	3.41	0.07	
						Interaction	0.59	0.44	
Comprehension	8.6 (0.5)	8.8 (0.4)		8.4 (0.5)	8.5 (0.5)	Group	2.07	0.16	.05
						Period	2.05	0.10	
						Interaction	0.17	0.68	
Expression	17.1 (2.0)	17.2 (1.7)		17.7 (1.3)	17.7 (1.2)	Group	1.20	0.28	.04
						Period	0.07	0.79	
						Interaction	0.07	0.79	
Remote memory	3.6 (1.9)	3.7 (1.6)		4.0 (1.8)	4.2 (1.5)	Group	0.55	0.46	.01
						Period	2.42	0.13	
						Interaction	0.20	0.65	
Recent memory	3.4 (0.8)	3.4 (0.6)		3.7 (0.6)	3.6 (0.6)	Group	1.31	0.26	.06
						Period	0.23	0.63	
						Interaction	1.36	0.25	
Learning memory	13.5 (1.7)	14.3 (1.2)		13.8 (2.0)	13.6 (1.6)	Group	0.12	0.72	.01
						Period	4.91	0.03*	
						Interaction	14.25	<0.01*	
Attention	4.6 (1.8)	4.6 (1.5)		5.2 (1.8)	4.9 (1.5)	Group	0.58	0.45	.03
						Period	0.97	0.33	
						Interaction	2.24	0.14	
Praxis	9.9 (0.8)	9.9 (0.8)		8.3 (0.9)	8.5 (0.9)	Group	25.05	<0.01*	.49^#^
						Period	1.46	0.23	
						Interaction	3.38	0.07	
Math	1.9 (0.2)	1.9 (0.2)		1.9 (0.3)	1.9 (0.3)	Group	0.01	0.89	.00
						Period	428.30	<0.01*	
						Interaction	0.00	1.00	
Abstract thinking	5.5 (1.8)	5.3 (1.6)		5.5 (1.1)	5.7 (1.0)	Group	0.19	0.66	.00
						Period	0.04	0.83	
						Interaction	5.07	0.03*	
Perception	9.1 (1.4)	9.0 (1.4)		8.4 (0.7)	8.3 (1.0)	Group	2.82	0.10	.09
						Period	0.38	0.54	
						Interaction	<0.01	0.95	
ABEP	15.2 (5.2)	15.8 (4.6)		16.3 (4.1)	17.0 (3.9)	Group	0.59	0.44	.02
						Period	8.40	<0.01*	
						Interaction	0.07	0.78	
Raven test	37.7 (8.0)	38.6 (7.4)		35.8 (5.2)	35.8 (4.8)	Group	0.95	0.34	.02
						Period	3.55	0.69	
						Interaction	3.55	0.69	
AUDIT	0.1 (0.5)	0.1 (0.5)		0.3 (1.0)	0.4 (1.3)	Group	0.41	0.53	.01
						Period	1.22	0.27	
						Interaction	1.22	0.27	
GDS-SF	2.2 (1.6)	2.0 (1.6)		2.2 (1.7)	3.2 (2.1)	Group	0.90	0.34	.00
						Period	2.23	0.14	
						Interaction	7.52	0.01*	

M: mean; SD: standard deviation; ABEP: Scale for assessment of Brazilian economic classification; Raven test: Raven’s Progressive Matrices; AUDIT: Alcohol Use Disorders Identification Test; GDS-SF: Geriatric Depression Scale, short form

(*) p<0.05;

(#) Eta-squared large effect: η2 >0.14.

There was no significant difference between groups across the CAMCOG subtests assessing episodic memory [F (1.29)=0.13, p=0.72], however, an effect for assessment period was noted [F (1.29)=4.91, p=0.03], i.e., a better performance by all participants was evident at the second assessment compared to the first. Additionally, there was an interaction effect between group and assessment period [F (1.29)=14.2, p<0.01].

A significant difference between the groups was found for praxis skills [F (1.29)=25.05, p<0.01] at both assessment periods, in that the active group performed better than the sedentary group. There was no significant difference in socioeconomic status between the groups. However, an effect for assessment period [F (1.29)=8.40, p<0.01] was detected by the *post hoc* Tukey test, i.e., an increase in the average economic level of all participants at the second assessment according to ABEP.^41^

No group [F (1.29)=0.91, p=0.35] or assessment period effects [F (1.29)=2.24, p=0.14] were found on the Geriatric Depression Scale though an interaction among these items [F (1.29)=7.53, p=0.01] was detected. Although the sedentary group, after one year, had higher scores on GDS-SF, the overall mean score of the group was less than 5 (cut off indicating depression). Notably, a year after the baseline assessment, one subject attained a score of over 5 on the GDS-SF (cut off recommended by the scale) in this group, i.e. symptoms indicative of mild depression.

There was a difference in self-report by the elderly for both groups regarding their ability to perform tasks involving memory usage, since the sedentary elderly had a lower sense of self-efficacy in relation to the active group. Results for the active group at baseline assessment were: Capacity (decrease 23.5%; maintained 58.8%; increase 17.7%), whereas after one year (decrease 23.5%; maintained 52.3%; increase 23.6%). The sedentary group at baseline assessment: Capacity (decrease 50.0%; maintain 42.8%; increase 7.2%) whereas after one year (decrease 57.1%; maintain 35.7%; increase 7.2%).

The total score difference in performance on the Memory Self-Efficacy Questionnaire (MSEQ) was not significant [F (1.29)=2.14, p=0.15] and there was no assessment period effect [F (1.29)=0.02, p=0.88]. However, there was a significant interaction [F (1.29)=7.07, p=0.01] in self-assessment of ability to perform tasks involving memory usage, especially long-term episodic memory, after one year.

On the longitudinal neuropsychological assessment - applied one year after the baseline assessment - a significant improvement was observed in the active group performance compared to the sedentary group on Free Words Recall, Visual Reproduction and Logical Memory tasks assessing episodic memory subtype performance and also on the scale for assessing activities of daily living (Table 2).

**Table 2:** Comparisons between performances by Groups on parameters assessed by neuropsychological assessment at baseline and after one year.

Parameters assessed	Active (n=17)			Sedentary (n=14)		Effects	F	p	η2
	Baseline assessment M±(SD)	After one year M±(SD)		Baseline assessment M±(SD)	After one year M±(SD)				
MSEQ	52.9 (11.9)	54.4 (10.9)		48.6 (12.3)	46.8 (10.7)	Group	2.14	0.15	0.03
						Period	0.02	0.88	
						Interaction	7.07	0.01*	
Free word recall immediate	5.9 (2.0)	5.4 (1.6)		4.2 (1.4)	5.4 (21.5)	Group	3.46	0.07	0.20^#^
						Period	0.04	0.83	
						Interaction	1.92	0.18	
Free word recall delayed	3.1 (2.0)	3.5 (1.3)		2.0 (1.2)	2.7 (1.4)	Group	6.45	0.01*	0.09
						Period	0.55	0.46	
						Interaction	9.25	<0.01*	
Visual Reproduction Immediate - A	4.8 (1.5)	5.2 (1.1)		4.6 (0.9)	4.1 (1.3)	Group	2.25	0.14	0.01
						4.8 (1.5)	0.55	0.46	
						Interaction	9.85	<0.01*	
Visual Reproduction Immediate - B	5.0 (1.9)	5.2 (1.5)		3.8 (1.8)	3.8 (1.7)	Group	4.23	0.04*	0.09
						Period	0.33	0.56	
						Interaction	1.84	0.18	
Visual Reproduction Immediate - C	7.5 (1.8)	7.3 (1.7)		6.1 (2.0)	5.7 (1.9)	Group	5.29	0.02*	0.12
						Period	5.30	0.02*	
						Interaction	0.44	0.51	
Visual Reproduction Immediate - D	13.6 (2.3)	13.8 (2.2)		12.7 (3.5)	12.1 (3.0)	Group	1.63	0.21	0.02
						Period	1.74	0.19	
						Interaction	6.25	0.01*	
Visual reproduction Delayed- A	4.1 (1.9)	4.6 (1.2)		3.6 (1.9)	3.1 (1.5)	Group	3.09	0.08	0.02
						Period	0.04	0.82	
						Interaction	4.28	0.04*	
Visual reproduction Delayed- B	4.4 (2.1)	4.7 (1.7)		3.2 (2.0)	2.7 (1.7)	Group	5.42	0.02*	0.07
						Period	0.23	0.63	
						Interaction	3.42	0.07	
Visual reproduction Delayed- C	6.9 (1.8)	6.2 (1.9)		5.9 (1.8)	5.2 (1.7)	Group	2.83	0.10	0.09
						Period	8.77	<0.01*	
						Interaction	0.06	0.79	
Visual reproduction Delayed- D	11.6 (3.4)	11.5 (2.9)		10.8 (3.6)	10.0 (2.9)	Group	0.98	0.32	0.01
						Period	2.38	0.13	
						Interaction	1.30	0.26	
Logical Memory Immediate	12.9 (3.1)	11.6 (2.3)		11.6 (2.9)	11.1 (2.5)	Group	3.50	0.07	0.05
						Period	0.24	0.62	
						Interaction	6.22	0.02*	
Logical Memory Delayed	11.2 (3.5)	10.0 (2.5)		9.1 (2.6)	8.7 (2.3)	Group	4.94	0.03*	0.10
						Period	1.27	0.26	
						Interaction	1.27	0.26	
Corsi Blocks: Forward	6.8 (1.3)	6.7 (1.4)		6.8(1.2)	6.6 (0.9)	Group	0.01	0.91	0.00
						Period	1.67	0.21	
						Interaction	1.67	0.21	
Corsi Blocks: Backward	5.0 (1.6)	5.1 (1.5)		5.5 (1.8)	5.1 (1.4)	Group	0.21	0.65	0.02
						Period	1.85	0.18	
						Interaction	3.23	0.08	
Digits - forward order	7.1 (1.1)	7.2 (1.1)		6.5 (1.2)	6.4 (1.2)	Group	2.60	0.11	0.06
						Period	0.07	0.79	
						Interaction	1.18	0.28	
Digits - backward order	4.6 (1.0)	4.8 (0.9)		4.4 (1.3)	4.3 (1.4)	Group	0.61	0.44	0.00
						Period	0.04	0.84	
						Interaction	3.67	0.06	
Fluency - F	10.1 (3.0)	10.2 (2.6)		10.9 (3.5)	10.9 (3.3)	Group	0.45	0.50	0.02
						Period	<0.01	0.96	
						Interaction	0.27	0.60	
Fluency - A	8.5 (2.1)	8.8 (1.9)		9.4 (2.8)	9.4 (2.7)	Group	0.79	0.38	0.03
						Period	0.47	0.49	
						Interaction	0.47	0.49	
Fluency - S	8.4 (2.0)	8.6 (1.7)		8.9 (3.0)	8.7 (2.9)	Group	0.14	0.71	0.01
						Period	0.11	0.73	
						Interaction	1.89	0.17	
Vocabulary	31.9 (5.2)	32.4 (4.7)		33.1 (6.2)	32.5 (5.7)	Group	0.10	0.74	0.01
						Period	0.34	0.56	
						Interaction	5.44	0.02*	
ADL	2.29 (1.0)	2.29 (1.2)		3.7 (1.6)	4.7 (1.7)	Group	17.14	<0.01*	0.24^#^
						Period	8.56	<0.01*	
						Interaction	8.56	<0.01*	

M: mean, SD: standard deviation; MSEQ: Memory Self-Efficacy Questionnaire; ADL: Index of Independence in Activities of Daily Living.

(*) p<0.05;

(#) Eta-squared large effect: η2 >0.14.

## DISCUSSION

The aim of the study was to determine possible changes in neuropsychological profile and the concept of self-efficacy of active and sedentary elderly women after 12 months by reassessing these parameters to investigate their relationship with physical activity.

For total score on the CAMCOG general cognitive screening test, there were no group or assessment-period effects.^40^ However, the significant interaction indicates an increase in scores over time for the active group and a slight decrease in scores for the sedentary group after one year. This interaction suggests that over the year, the general cognitive performance was better in the group of elderly practicing regular physical activity,^23-25^ showing that participation in physical activity programs benefits the physical and psychological spheres and that physically active individuals most likely have faster cognitive processing.^18,19^ This suggests that physical activity may be an important protective factor against cognitive impairment and dementia in elderly.^26,27^

However, the scores on subtests of the CAMCOG for episodic memory tasks indicate improvement in performance on learning memory (Table 1) by all participants in the active elderly group after one year compared to baseline assessment. Physical activity might have positively affected the scores, but differences were not detected by Tukey's *post hoc* test. These were confirmed however, on the neuropsychological tests, one year after the baseline assessment by specific tests for episodic memory, mainly in delayed recall (Table 2). The active group also had a higher degree of self-efficacy for the tasks involving higher memory capacity.^13,17^ This provides indirect evidence that regular practice of physical activity is associated with an improvement in motivation and sense of self-efficacy.^20,21^

In the evaluation of praxis by the CAMCOG, the active group performed better than the sedentary group at both assessment periods (baseline and after one year). There was a positive association with the scores obtained on the Index of Independence in Activities of Daily Living, in which the active elderly performed significantly better than the sedentary group. This association indicates that the limitations in praxis of the sedentary group interfere in daily living.^18,19,23-25^

Despite significant differences between the active and sedentary elderly on tasks involving episodic memory skills and praxis, there were no group differences for vocabulary, phonological and semantic verbal fluency or working memory (Table 1), consistent with the notion that episodic memory decline prevails over semantic memory impairment in the non-pathological aging process.^13^

The Memory Self-Efficacy Questionnaire (MSEQ) revealed an interaction between performance by group and assessment period, showing an increase in scores after one year for the active group and a slight decrease in scores for the sedentary group in the self-assessment of ability to perform tasks involving memory usage. Considering that individuals' perceptions of their efficacy affect the projections and predictions about the outcome of their actions, it follows that a negative evaluation of self-efficacy for memory predicts failure while a positive evaluation predicts success.^17^

With regards to mood states, no difference in the performance of each group for scores on the GDS-SF was evident, as there was no assessment period effect. Nevertheless, there was a significant interaction, which indicates an increase in scores after one year in the sedentary group and a slight decrease in scores for the active group, indicating greater depressive symptoms in the former group and fewer in the latter after one year. Thus, these results suggest that the mood of active elderly was significantly better than that reported by the sedentary women.

Although some cognitive functions are negatively affected by age due to the loss of neurons concomitant with decline in cognitive performance,^6^ the processes based on crystallized abilities, such as verbal knowledge and comprehension, remain unaffected or improve with aging. On the other hand, procedures based on fluid abilities, such as learned tasks but not implemented tasks, may suffer decline.^7^ This fact is evidenced by the results obtained in our study, as significant differences were found, revealing better performance of active elderly compared to sedentary elderly on activities involving skills, episodic memory and praxis, but not tasks of vocabulary, verbal, semantic and phonological fluency or working memory.

Regarding limitations of this study, the low number of participants should be considered. Convenience sampling was used, however, the ETA-squared index showed medium and large effect sizes for most of the dependent variables. Another issue of concern may be the time elapsed between baseline and follow-up assessments. However, based on previous studies we may assume that a one-year follow-up period is a reliable interval.^12,28^ In addition, we detected cognitive changes in this short timeframe, which may serve as valuable information for rehabilitation purposes.

In summary, active elderly exhibited superior performance compared to the sedentary group with regards to episodic memory, praxis skills and mood state. The practice of regular physical activity accompanied a greater sense of self-efficacy. Therefore, the results of this longitudinal study suggest that regular physical activity may be an important protective factor against cognitive impairment, dementia and depression in the elderly,^26,27^ and serve as an early intervention in order to reduce these effects.^14,36,37^
